# Posttraumatic Displacement Management: Lateral Luxation and Alveolar Bone Fracture in Young Permanent Teeth with 5 Years of Follow-Up

**DOI:** 10.1155/2015/634237

**Published:** 2015-03-08

**Authors:** Heitor Marques Honório, Catarina Ribeiro Barros de Alencar, Edmer Silvestre Pereira Júnior, Daniela Silva Barroso de Oliveira, Gabriela Cristina de Oliveira, Daniela Rios

**Affiliations:** ^1^Department of Pediatric Dentistry, Orthodontics and Public Health, Bauru School of Dentistry, University of São Paulo, Alameda Dr. Octávio Pinheiro Brisolla 9-75, P.O. Box 73, 17012-101 Bauru, SP, Brazil; ^2^Department of Pediatric Dentistry, Federal University of Alfenas, Rua Gabriel Monteiro 700, 37130000 Alfenas, MG, Brazil

## Abstract

Dental trauma is an important public health problem due to high prevalence and associated limitations. The external impact accounting for trauma may result in different injury types to teeth and supporting structures. This paper describes a clinical case of tooth trauma in an 8-year-old patient exhibiting the displacement of three permanent teeth with open root apexes. Although the traumatic impact resulted in two injury types to teeth and supporting tissues (lateral luxation and alveolar bone fracture), the therapeutic approach was the same in both situations. The bone and teeth were repositioned by digital pressure, stabilized by semirigid splint, and followed up at every week. After six weeks, the splint was removed. At that moment, the clinical and radiographic findings indicated normal soft/hard tissues and absence of pulp/periodontal pathologies. At the fifth year of follow-up, the treatment success of the case was confirmed, although it has been observed that all lower incisors exhibited pulp obliteration as a consequence of the dental trauma.

## 1. Introduction

Dental trauma is an important public health problem, due to high prevalence, especially among children and teenagers [1]. Additionally, it negatively impacts on the person's quality of life [2] because of the esthetic, psychological, social, functional, and therapeutic problems [3].

An external impact causes the traumatic tooth lesions, which may result in different injury types to teeth and supporting structures. Lateral luxation is the term used to describe tooth displacement towards a direction different from axially [4], followed by alveolar bone fracture in only one side of the alveolar bone (either labial or lingual/palatal). If both alveolar socket sides have been fractured, the injury should be classified as alveolar fracture, characterized by the involvement of multiple teeth and alveolar process mobility with movement as a unit of the displaced segment [5].

Because of the neurovascular bundle displacement and periodontal ligament damage occurring in the traumatic situations, posttraumatic healing will be implied in pulp revascularization/reinnervation and periodontal fiber reorganization/recovery. In teeth exhibiting immature root development, the repair may occur through developing either new blood vessels in pulp chamber or blood vessel anastomosis in apical area [6].

According to the “dental trauma internet calculator” at the Dental Trauma Guide (http://www.dentaltraumaguide.org), only 7 teeth from 4 patients have been recorded as undergoing alveolar fracture. Considering that the literature has reported few cases of lateral luxations and alveolar fractures in young permanent teeth, the aim of this study was to report the association of these two trauma types in permanent teeth with open apexes, in which it was possible to perform immediate treatment and 5-year follow-up.

## 2. Case Report

**Figure 1:**
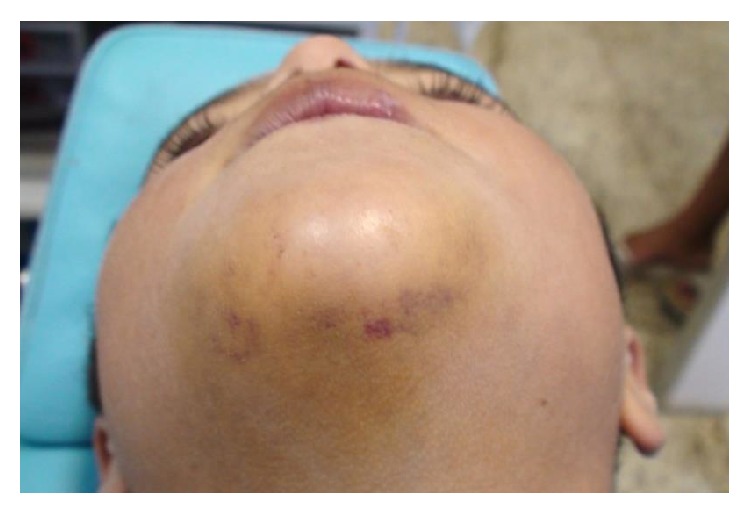
Extraoral view showing contusion lesion and abrasion on the chin.

**Figure 2:**
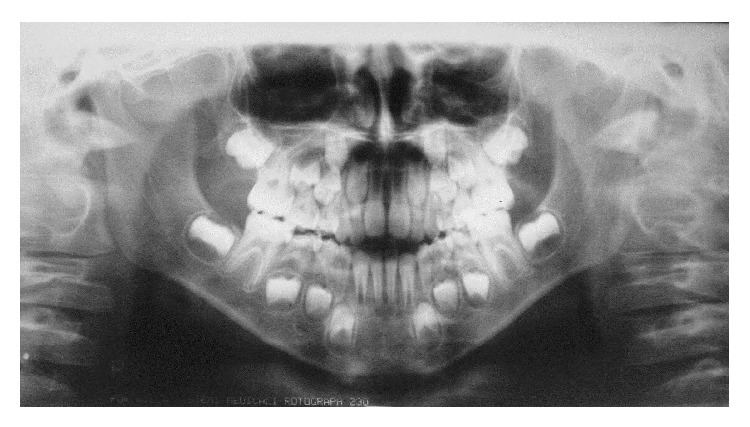
Panoramic radiograph. Normal TMJ structures, without injury.

A male patient, aged 8 years, suffered a traumatic injury in the area of the chin and mandibular right and left central incisor and mandibular right lateral incisor after he had been pushed against a wall by a friend at school. The child was referred to the dentist one hour after trauma, reporting intense pain in the traumatic area. At extraoral clinical examination, the presence of contusion lesion and abrasion on the chin was observed (Figure 1). Considering the traumatic impact on the chin and the possible damage to the temporomandibular joint (TMJ), a panoramic radiograph was taken to evaluate this area. The radiographic image indicated normal TMJ structures, without injury (Figure 2). Thus, extraoral injuries treatment was restricted to the cleaning and disinfection of the damaged soft tissue.

**Figure 3:**
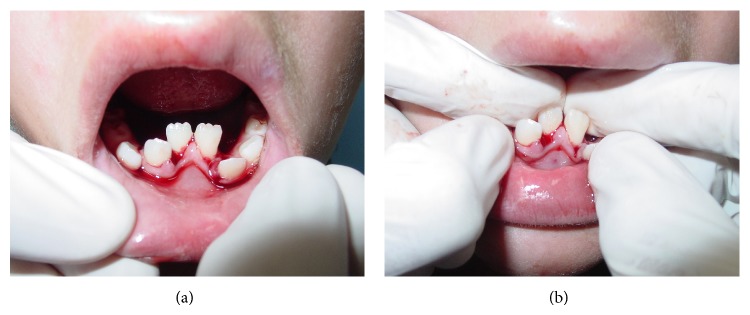
(a) Initial intraoral view. (b) Bone fracture reduction by digital pressure.

Intraoral examination revealed the presence of lateral luxation towards the lingual surface of mandibular right lateral incisor (without mobility) and alveolar fracture of the area of teeth mandibular right and left central incisor with lingual displacement (Figure 3(a)). When mobility was checked, alveolar process showed movement as a unit of the displaced segment, which characterizes the alveolar bone fracture. The alveolar fracture was not visualized on radiographs, since they were taken only after dental trauma treatment. The preoperative radiograph was not taken because the child was in pain and the image was not necessary for the choice of treatment.

**Figure 4:**
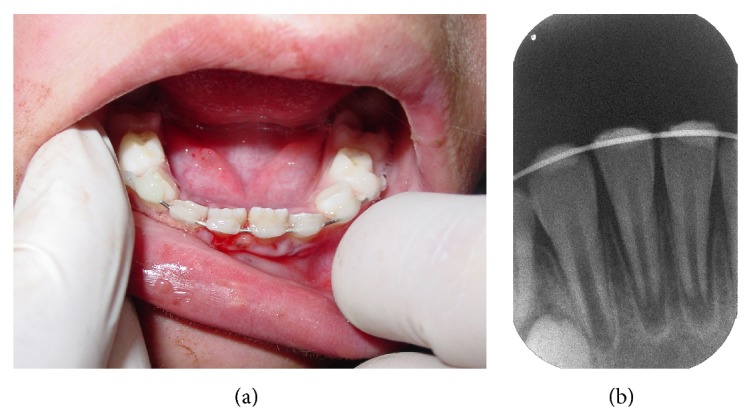
(a) Flexible splint made with resin composite and 0.7 mm orthodontic wire onto the labial surface of the teeth involved and the immediately adjacent teeth. (b) Radiographic of the injured area.

Although the traumatic impact caused two injury types to teeth and supporting tissue (lateral luxations and alveolar fracture), the management performed was the same for both situations. After anesthesia with local and intraligamentary infiltration of the displaced teeth, using a full cartridge of 2 percent lidocaine with 1 : 100,000 epinephrine, the mandibular right lateral incisor and the bone segment of mandibular right and left central incisor were repositioned by digital pressure. The bone fracture reduction was difficult to execute because of its extension (Figure 3(b)). Next, a splint made with resin composite (Filtek Z350 XT, 3M ESPE) and 0.7 mm orthodontic wire was placed onto the labial surface of the teeth involved and the immediately adjacent teeth (not injured) (Figure 4(a)). At the periapical radiographic examination of the injured area, the image showed the presence of open axes in the three teeth affected by trauma (Figure 4(b)).

The patient was instructed about oral hygiene and the importance of follow-up appointments. The use of 0.12% chlorhexidine solution for application on injured site was recommended, twice daily during the first week after the dental trauma. Paracetamol was prescribed to be used while the patient was in pain and was used only in the first day (24 h). Amoxicillin was prescribed for use during 7 days, once the child fell in school, a possibly contaminated area.

**Figure 5:**
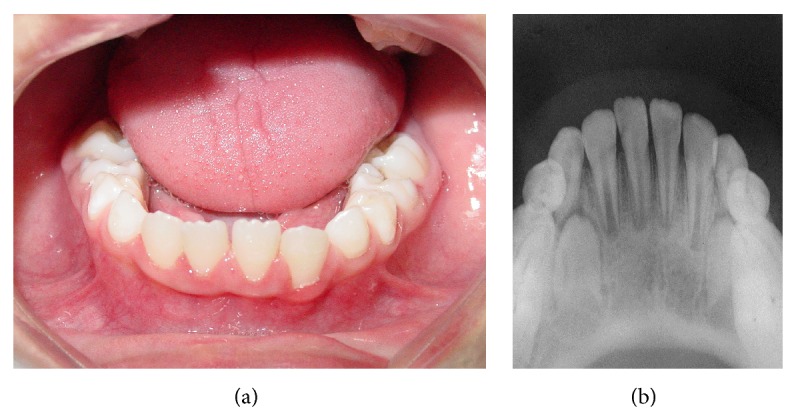
(a) Clinical aspect after removal of the splint showing the normality of soft tissues. (b) Radiographic image without splint (after 6 weeks of the trauma) indicating no pulp and periodontal pathologies.

**Figure 6:**
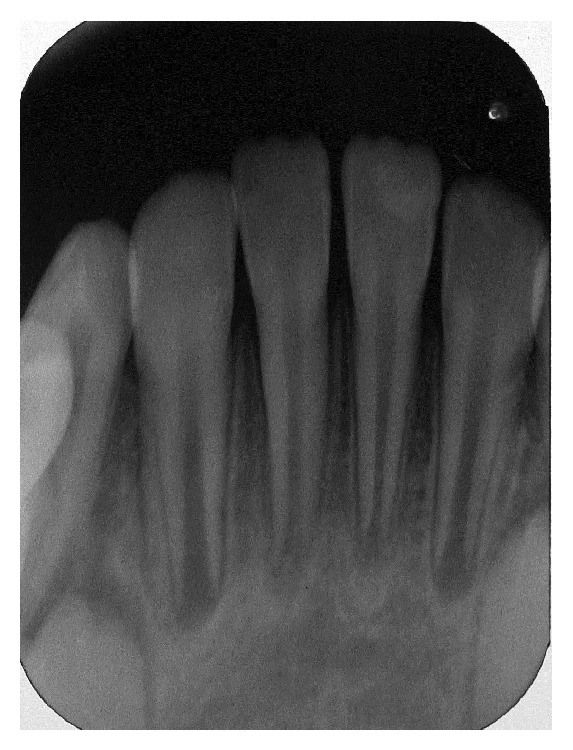
Six-month radiographic follow-up. Closure process of the apexes of the traumatized teeth continued normally.

**Figure 7:**
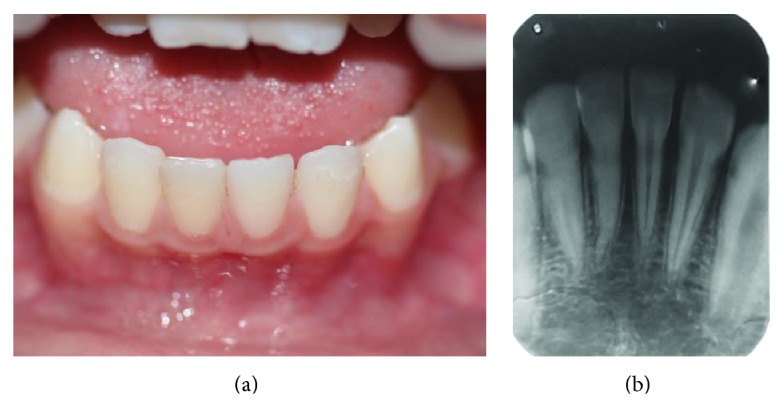
(a) Five-year clinical follow-up. (b) Radiographic image after 5 years of the trauma. Full closure of the apexes and marked obliteration of the root canal lumens.

After one week, the splint loosened probably because of the difficulty in controlling the moist from the injured tissue at the emergency appointment and failure of the bonding system of the resin composite. The splint was again installed because the teeth still showed mobility. The patient was followed up at every week. After 6 weeks, the splint was removed and the clinical (Figure 5(a)) and radiographic (Figure 5(b)) findings indicated normality of soft and hard tissues with no pulp and periodontal pathologies. At three months after trauma, the patient was followed up at every two weeks, followed by two-month intervals to monitor the teeth involved in the trauma. After six months, the patient returned with a broken leg due to fall, but without involving the maxillofacial area. The follow-up radiographic examination evidenced the continuity of the closure process of the apexes of the traumatized teeth (Figure 6). The 5-year follow-up indicated the presence of normal clinical aspects (Figure 7(a)). All teeth exhibited pulp vitality to Endo Frost Roeko cold spray test. Radiographically, the full closure of the apexes followed by a marked obliteration of the root canal lumens could be verified (Figure 7(b)).

## 3. Discussion

Dentoalveolar injuries are common [4], especially among children and teenagers at dental and facial development period [7]. These traumas may affect both the primary and permanent dentition. Notwithstanding, trauma to periodontal supporting tissues (tooth luxations and avulsion) occurs more often on primary teeth, while trauma to hard tissues (crown, root, and crown-root fractures) has been more frequently observed on permanent teeth [8].

Due to more medullar than cortical bone, the greatest elasticity of alveolar bone accounts for the periodontal involvement frequently seen in traumas to primary teeth [9]. Accordingly, the greater resilience capacity of alveolar bone and periodontal ligament together with the smaller clinical crown and proportionally shorter root allows the absorption of the traumatic impact, favoring the displacements in comparison to fractures. With aging, although the impact type is not altered, the bone resilience decreases, so that the impact will be normally on the tooth itself [1].

In this present case, the trauma affected the periodontal supporting tissue (lateral luxations and alveolar fracture). Given the fact that the patient had 8 years old at the moment of the trauma, it can be assumed that the characteristics of alveolar bone resilience were probably not completely modified. Additionally, the incomplete root formation of the teeth involved helps explain the trauma nature due to the less involvement of the root portion, enabling the traumatic impact stress dissipation on the tissue surrounding the teeth.

The literature points out that falls are the major cause of dental and maxillofacial traumas [1]. In the case reported, the patient did not fall, but he had probably inclined his head backwards at the moment of pushing against the wall, causing the impact on the chin. Additionally, during the impact, his mouth was opened and the mandible projected forwards, leading to the involvement of the mandibular teeth and displacement of the anterior inferior segment lingually.

Displaced and luxated teeth undergo damage to pulp and periodontium [10]. Nevertheless, the immature permanent tooth has great capacity of posttraumatic healing [11] and the prognosis is favorable even with late repositioning [12]. However, to postpone the treatment can make adequate tooth positioning difficult due to the presence of organized blood clot inside alveolar socket. Thus, immediate repositioning [13] enables the faster and less costly resolution of the problem, fulfilling with the treatment goals of dentoalveolar injuries: to restore the occlusion function, reestablish the esthetics, and optimize the development of dentition, growth of jaws, and surrounding soft tissues [7].

The stabilization of injured teeth through using the adjacent sound teeth is considered the best practice to support the tooth at right position and in function because it allows the exposure of the injured teeth to physiologic forces existing in oral environment. Moreover, the stabilization either reduces or avoids pain, offers comfort to patient, and protects the teeth from traumatic forces during healing process [14].

Over the last decades, the knowledge on the repair of teeth traumatically displaced was improved and treatment guidelines have been more based on evidence [15]. For example, longer splint periods and rigid splints increase the risk of healing complications [16]. Accordingly, flexible splints [17] for shorter periods are more effective [18] while the mechanical stimulus exerted by the light movement of the teeth favors the revascularization process and is capable of preventing tooth ankylosis and maintaining the vitality of Hertwig's epithelial root sheath [19], which is essential in developing roots [18]. The splint period for periodontal ligament therapy is 2–4 weeks, but in case of either lack of periodontal support or marginal bone weakening, as in this present case, the ideal period should be postponed for until 8 weeks [20].

Many splint types have been used in daily practice, but regardless of the type, passivity and flexibility are essential features to promote bone reestablishment and periodontal ligament fiber rearrangement [14]. The splint made from orthodontic wire and resin composite to stabilize traumatically displaced teeth, as performed in this present case, has the advantage of using low-cost materials generally available in dental offices [21]. Also it leads to satisfactory outcomes because the characteristics decrease the risks of complications such as ankylosis, root resorption, and pulp obliteration [22].

Although it is not possible to avoid completely accidents resulting tooth traumas, immediate first-aids and proper follow-up can prevent complications [23]. Moreover, the patients and parents should be instructed about the importance of new tooth lesion prevention, for example, avoiding participating in contact sports [4]. The fact that the patient of this case report returned with a broken leg at one of the appointments confirmed the risk for occurrence of new tooth traumas because of biologic features, such as gender (males), age range (8 to 10 years), and energetic behavior (tendency towards more vigorous activities) [1].

Studies evaluating the occurrence of consecutive tooth traumas point out that almost at every second, 8–18-year old patients suffering tooth trauma are at risk of undergoing new trauma episodes on the traumatized teeth by more than 50% [24]. Many traumatic episodes on the same teeth increase the possibility of lesion worsening, resulting in greater risk of developing future complications and increasing treatment costs due to the necessity of treatment replanning [25].

Major complications related to severe dental traumas are the replacement resorption and ankylosis. The absence of vital periodontal ligament in substantial areas of root surface may enhance resorption of the cementum and dentin by osteoclasts from the adjacent bone marrow. The resorbed tooth dentin is replaced with alveolar bone by osteoblasts [26]. The ankylosis of a permanent incisor in children and adolescent might result in inevitable early loss of the traumatized tooth and local arrest of alveolar bone [27]. After 5 years of the dental trauma, the patient of the present case did not develop radiographic aspects (disappearance of periodontal ligament width and the replacement of root dentin with bone) or clinical signs of ankylosis. In addition, infraposition of the injured teeth was not observed.

At the 5-year clinical follow-up the teeth also exhibited pulp vitality to cold spray test. Radiographically, the teeth showed properly closure of root apexes and severe root obliteration. Calcification is a pulp tissue complication following traumatic teeth displacement [10]. In the future, if these teeth require endodontic intervention, the pulp obliteration can be a relevant factor that might hamper the treatment.

Based on the clinical case reported here, it can be concluded that although lateral luxation associated with alveolar fracture of young permanent teeth initially compromises patient's esthetics, function, and well-being, the immediate treatment results in good prognosis, with minor complications.
